# Case Report: Co-occurrence of Wilson disease and tuberous sclerosis complex in a Chinese patient

**DOI:** 10.3389/fgene.2026.1855858

**Published:** 2026-09-25

**Authors:** Kun Xia, Shijing Wang, Tong Wu, Dandan Sun, Wei Wang, Xun Wang, Yongsheng Han

**Affiliations:** 1 Affiliated Hospital of the Institute of Neurology, Anhui University of Chinese Medicine, Hefei, Anhui, China; 2 Wannan Medical University, Wuhu, Anhui, China

**Keywords:** *ATP7B* mutation, case report, *TSC2* mutation, tuberous sclerosis complex, Wilson disease

## Abstract

**Background:**

Wilson disease (WD) and tuberous sclerosis complex (TSC) are rare genetic disorders with distinct pathophysiologies. Their co-occurrence has not been previously reported. When atypical clinical features emerge in a patient with an established genetic diagnosis, comprehensive reassessment, including thorough physical examination and expanded genetic testing, becomes critical.

**Case Presentation:**

We describe a 28-year-old Chinese woman with a confirmed diagnosis of WD who developed focal seizures after substantial clinical improvement during copper chelation therapy. Her seizures were controlled with carbamazepine (600 mg/day), but repeated attempts to withdraw carbamazepine led to seizure recurrence, prompting diagnostic reassessment. Physical examination revealed characteristic TSC stigmata, including facial angiofibromas, periungual fibromas, a shagreen patch, and hypomelanotic macules. Laboratory evaluation demonstrated markedly low serum copper (1.38 μmol/L) and ceruloplasmin (65.0 mg/L), along with elevated 24-h urinary copper excretion (218.20 μg/24 h), measured during penicillamine treatment. Imaging showed nodular liver changes, a left renal hamartomatous lesion, bilateral basal ganglia abnormalities, a probable cortical tuber, and subependymal nodules. In January 2025, whole-exome sequencing identified a homozygous pathogenic *ATP7B* variant (NM_000053.4: c.2333G>T; p.Arg778Leu), confirming WD, and an apparently *de novo* heterozygous pathogenic *TSC2* frameshift variant (NM_000548.5: c.2816dup; p.Ser939ArgfsTer21). The *TSC2* variant was absent in both tested parents, but biological parentage was not independently confirmed.

**Conclusion:**

To our knowledge, this is the first reported case of concomitant WD and TSC. Given their distinct genetic etiologies, including a *de novo* TSC2 variant in this patient, the co-occurrence is most appropriately interpreted as an exceptionally rare independent dual diagnosis rather than evidence of a biological link. The case highlights diagnostic overshadowing and the importance of renewed physical examination and expanded genetic testing when the clinical course becomes atypical for an established disorder.

## 1 Introduction

Wilson disease (WD) is a rare, autosomal recessive disorder of copper metabolism, caused by pathogenic variants in the *ATP7B* gene located on chromosome 13q14.3. The global incidence is estimated at 1/45,000–1/35,000 (Lucena-Valera et al., 2023); more recent data from large-scale genetic studies suggest a frequency of 1/7,026 (Ovchinnikova et al., 2024). The pathological features of WD include impaired ceruloplasmin synthesis and defective cellular copper transport, leading to toxic copper accumulation in organs such as the liver and basal ganglia. The clinical presentation of WD encompasses common features such as cirrhosis, neuropsychiatric disorders, and Kayser-Fleischer rings, with cutaneous changes and seizures being rare (Dzieżyc et al., 2017; Rao et al., 2018).

Tuberous sclerosis complex (TSC) is an autosomal dominant neurocutaneous syndrome caused by germline variants in either the *TSC1* or *TSC*2 gene. These variants lead to hyperactivation of the mechanistic target of rapamycin (mTOR) signaling pathway, resulting in the formation of multiorgan hamartomas. The disease affects approximately 1 in 6,000 individuals and is characterized by clinical features such as facial angiofibromas, epileptic seizures, subependymal nodules, and visceral hamartomas (Wang et al., 2021).

The co-occurrence of WD and TSC is exceptionally rare due to their distinct genetic bases and low individual prevalence. Neurological symptoms such as epilepsy, which can appear in both conditions, complicate diagnosis when they overlap. We performed a systematic literature search in PubMed and Web of Science using the search terms (“Wilson disease” OR “hepatolenticular degeneration” OR “*ATP7B*”) AND (“tuberous sclerosis complex” OR “*TSC*” OR “*TSC2*”), without language restrictions and covering all records available up to January 1, 2026. This search yielded no reports of coexistent WD and TSC in the same patient. Here, we present such a patient confirmed by clinical and genetic assessments, emphasizing the clinical problem of diagnostic overshadowing and the value of detailed re-examination and expanded genetic testing when new findings do not fit an established diagnosis.

## 2 Case description

A 28-year-old woman presented with a 15-year history of dysarthria and hand tremors. Her symptoms began at age 13 in 2009, with dysarthria and involuntary hand tremors, leading to a diagnosis of WD based on low serum ceruloplasmin (80.0 mg/L), elevated 24-h urinary copper excretion (358 μg/24 h), and reported symmetric bilateral lentiform nucleus lesions typical of WD on brain MRI. The original 2009 MRI images and other early imaging records are unavailable, limiting retrospective verification of the initial radiological findings. The patient was treated with dimercaptopropane sulfonate for copper chelation. After discharge, she received long-term penicillamine (750 mg/day) treatment, with substantial improvement of dysarthria and tremors.

At age 16 (2012), she experienced recurrent episodes of left-sided limb convulsions with impaired awareness. The 2012 brain MRI reportedly showed abnormal signals in the bilateral lentiform nuclei and right frontal cortex, but the original images are unavailable. The contemporaneous routine EEG reportedly showed sharp-wave discharges over the right frontocentral and frontoparietal regions; neither the original EEG tracing nor the original report is available. Thus, the epilepsy was initially attributed to WD, and carbamazepine treatment was started. Her seizures remained controlled at a dosage of 600 mg/day; however, attempts to withdraw carbamazepine at ages 25 (2021) and 27 (2023) led to seizure recurrence, necessitating continued treatment. The patient was readmitted for further evaluation in December 2024. Her medical and family histories were unremarkable. (see the timeline in Figure 1).

**FIGURE 1:**
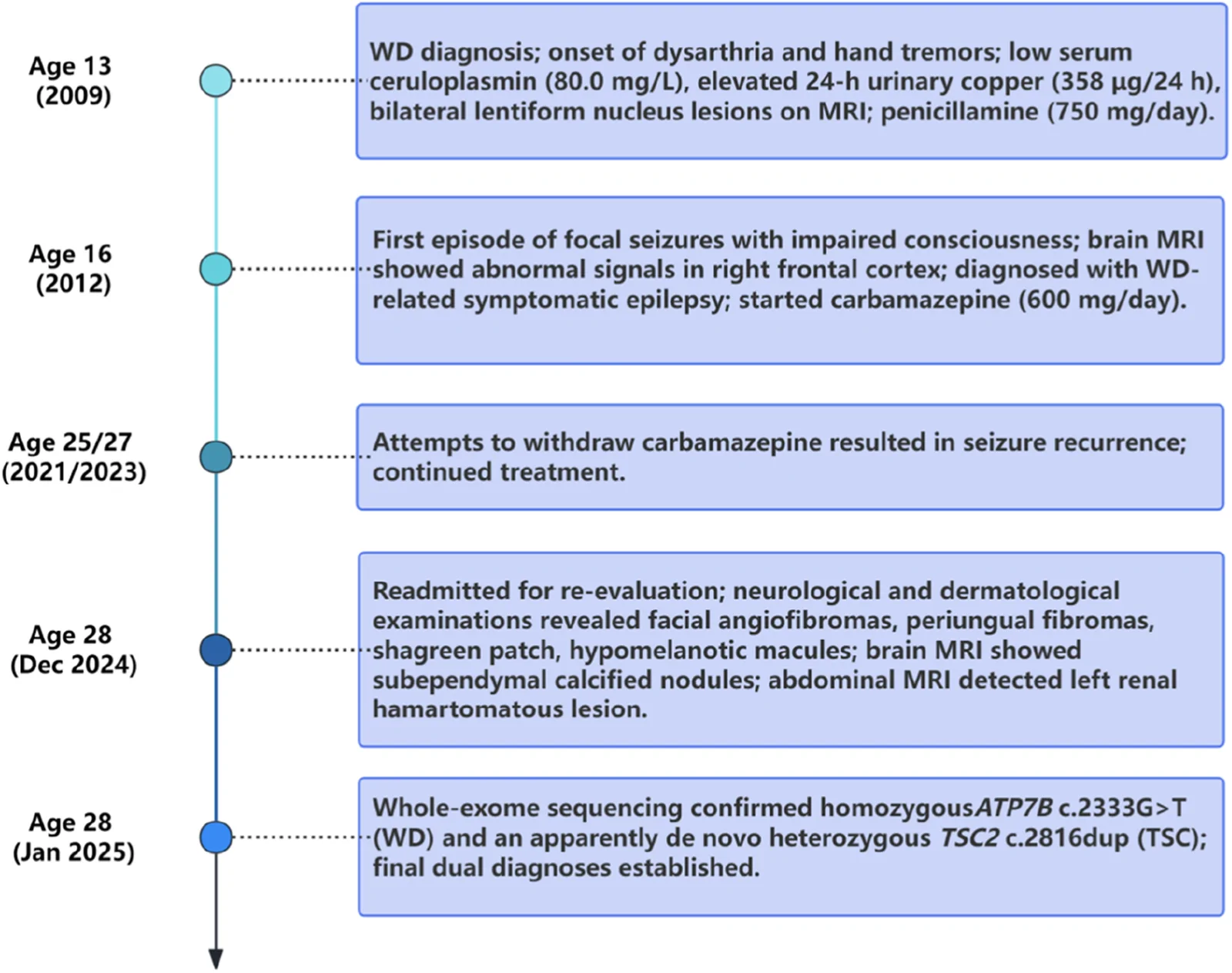
The patient’s course and associated diagnosis including signs, symptoms, and test results are noted on the timeline.

Neurological examination upon readmission revealed mild dysarthria and high-frequency postural tremors in both upper limbs. Muscle tone was mildly increased, with lead-pipe rigidity in all limbs. Coordination testing demonstrated clumsiness during rapid alternating movements. The Montreal Cognitive Assessment score was 28/30. Dermatological examination revealed symmetrically distributed brown papules in the nasolabial folds (facial angiofibromas), periungual fibromas on several digits, a shagreen patch in the lumbosacral region, and multiple hypomelanotic macules (Figure 2A).

**FIGURE 2:**
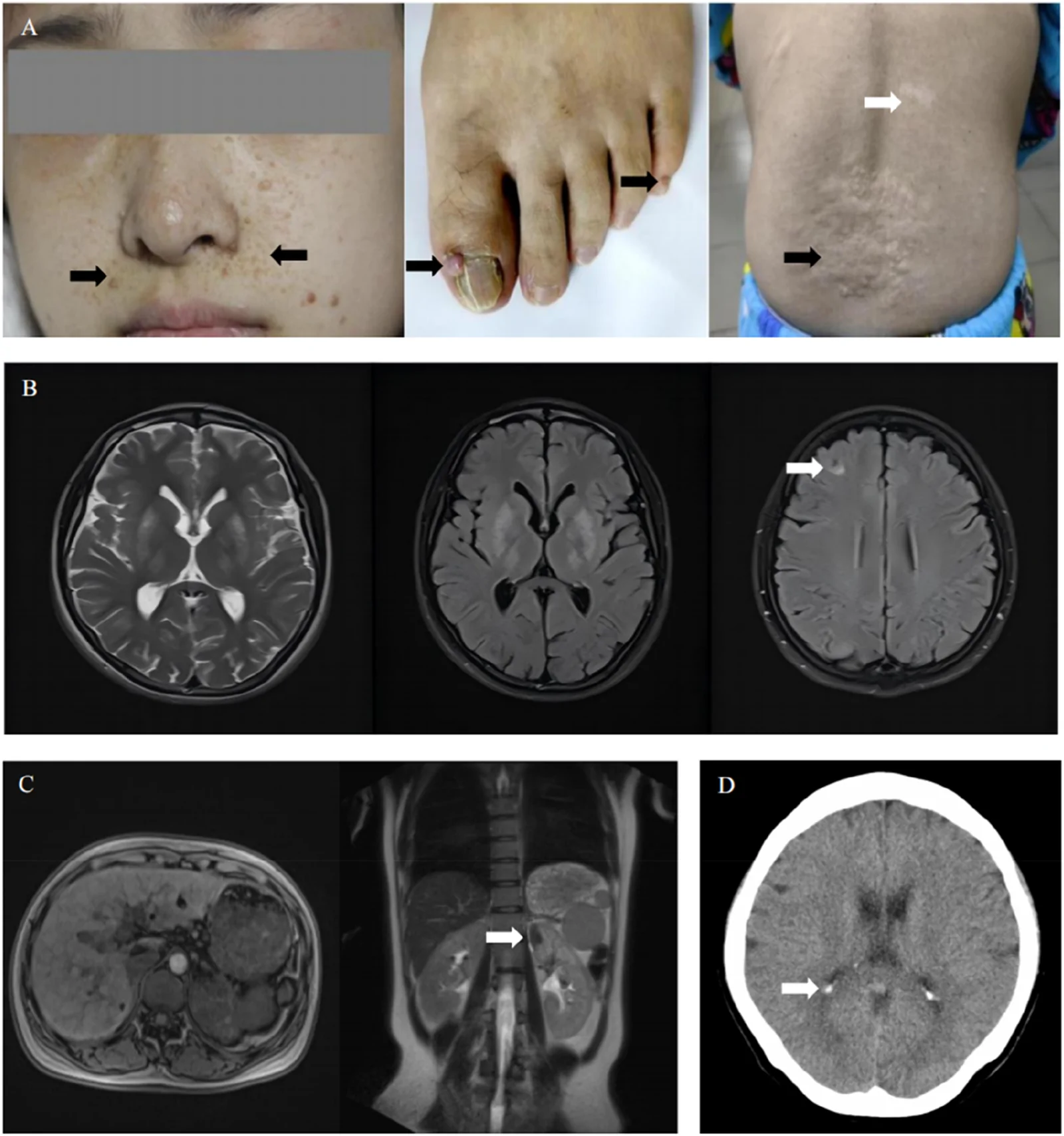
**(A)** Cutaneous findings: facial angiofibromas presenting as symmetrically distributed brown papules in the nasolabial folds, periungual fibromas, and a shagreen patch (black arrow) with hypomelanotic macules (white arrow) on the lumbosacral skin. **(B)** Brain MRI: axial T2-weighted and FLAIR images demonstrate symmetric hyperintensities in the lentiform nuclei, compatible with WD; axial T2-weighted imaging shows a focal cortical signal abnormality in the right frontoparietal lobe (arrow). **(C)** Abdominal MRI: T1-weighted imaging shows nodular hepatic parenchyma, consistent with WD-related cirrhosis; T2-weighted imaging shows a left renal hamartomatous lesion (arrow). **(D)** Cranial CT: bilateral subependymal calcified nodules are visible (arrow). All images in this figure were obtained for the present report. Prior MR images from the initial evaluation were unavailable for comparison.

Given the critical importance of copper metabolism indices for the diagnostic evaluation of WD, the relevant tests were repeated and confirmed, revealing low serum copper levels (1.38 μmol/L; reference range 11–24 μmol/L), low ceruloplasmin levels (65.0 mg/L; normal range 200–600 mg/L), and elevated 24-h urinary copper excretion (218.20 μg/24 h; normal value < 100 μg/24 h).

Brain MRI revealed persistent T2/FLAIR hyperintensities in both lentiform nuclei (Figure 2B), supporting the diagnosis of WD. Additional findings on brain MRI included a focal cortical signal abnormality in the right frontoparietal lobe (Figure 2B) which was radiologically consistent with a cortical tuber, although confirmatory fluid-attenuated inversion recovery (FLAIR) sequence at the time did not demonstrate a definitively characteristic transmantle sign. Abdominal MRI confirmed nodular liver morphology and a left renal hamartomatous lesion (Figure 2C). Cranial CT showed bilateral basal ganglia hypodensity and characteristic subependymal calcified nodules (Figure 2D). Kayser-Fleischer rings were not detected. Echocardiography and chest CT findings were unremarkable. Furthermore, a 24-h ambulatory EEG performed while the patient was receiving carbamazepine (600 mg/day) revealed no epileptiform discharges.

In January 2025, the patient underwent whole-exome sequencing. Genetic analysis identified a homozygous missense variant in exon 8 of the *ATP7B* gene (NM_000053.4: c.2333G>T; p.Arg778Leu), which was classified as pathogenic and confirmed the molecular diagnosis of WD (Figures 3A–C). Sanger sequencing showed that both tested parents were heterozygous carriers of the *ATP7B* c.2333G>T variant (Figures 3B,C), consistent with the autosomal recessive inheritance. p.Arg778Leu is a common pathogenic *ATP7B* variant in East Asian WD populations (Ovchinnikova et al., 2024) and was not interpreted as evidence of variant rarity or parentage. Formal STR-based parentage testing was not performed. A previously unreported heterozygous frameshift variant in exon 25 of *TSC2* (NM_000548.5: c.2816dup; p.Ser939ArgfsTer21) was identified (Figure 3D); it was absent from the gnomAD East Asian population database and unreported in ClinVar at the time of analysis. Parental testing and Sanger sequencing showed that the variant was absent in both tested parents (Figures 3E,F), consistent with an apparently *de novo* event. Because parental relationships were not independently confirmed, the *de novo* evidence was conservatively weighted as PS2_Moderate rather than full PS2. The overall mean target sequencing coverage was 137.53×, with approximately 130× coverage at the variant locus. The frameshift is predicted to cause nonsense-mediated mRNA decay and was classified as pathogenic under ACMG/AMP criteria (PVS1, PS2_Moderate, and PM2_Supporting).

**FIGURE 3:**
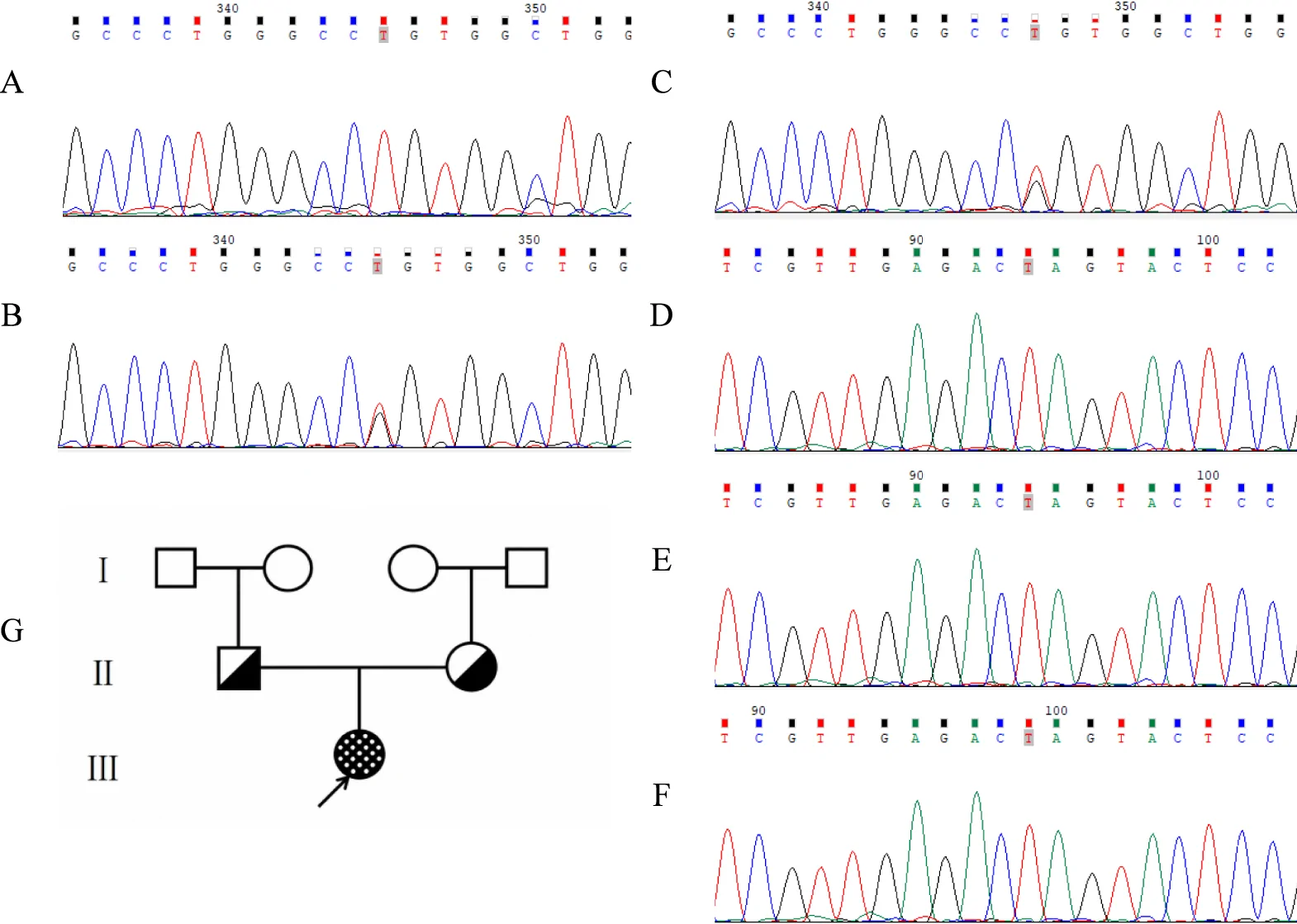
**(A–C)** *ATP7B* analysis: **(A)** the proband harbored a homozygous p.Arg778Leu variant inherited from both the **(B)** father and **(C)** mother. **(D–F)** *TSC2* analysis: **(D)** the proband carried a heterozygous p.Ser939ArgfsTer21 frameshift variant that was **(E,F)** absent in both tested parents, consistent with an apparently *de novo* event; biological parentage was not independently confirmed. **(G)** Family pedigree. Half-shading denotes heterozygous carrier status for the ATP7B variant, and stippling denotes the *TSC2* variant. The proband is homozygous for the ATP7B variant and heterozygous for the *TSC2* variant; both parents are heterozygous *ATP7B* carriers and tested negative for the *TSC2* variant. All Sanger sequencing chromatograms were generated from this study’s own analyses.

The *TSC2* finding prompted careful phenotypic review. Lymphangioleiomyomatosis (LAM) was considered unlikely because chest CT findings were normal and the patient had no respiratory symptoms. Isolated type II focal cortical dysplasia (FCD II) could account for the focal epilepsy and cortical lesion but not the characteristic cutaneous and renal findings. TSC therefore provided the most coherent multisystem diagnosis.

The final diagnoses were established using validated scoring systems and consensus criteria. For WD, the Leipzig scoring system (European Association for the Study of the Liver, 2025) (where a score ≥4 indicates a definite diagnosis) yielded 9 points: (1) mild dysarthria and postural tremors (1 point); (2) a serum ceruloplasmin concentration <100 mg/L (2 points); (3) a 24-h urinary copper concentration >200 μg (2 points); and (4) a homozygous pathogenic variant in exon 8 of *ATP7B* (4 points). The 24-h urinary copper measurement was obtained during penicillamine therapy, which increases urinary copper excretion and therefore confounds this component; even if this component were excluded, the Leipzig score would remain 7, above the diagnostic threshold of 4. For TSC, the patient met the 2021 International TSC Consensus Conference diagnostic criteria (Northrup et al., 2021) on clinical grounds, with at least four definite major features: facial angiofibromas, periungual fibromas, a shagreen patch, and subependymal nodules. Hypomelanotic macules were also present; however, the available record did not document the number and size required to count them as a separate major feature. Brain MRI showed a right frontoparietal cortical lesion compatible with a probable cortical tuber, but this finding was not required for the diagnosis. A heterozygous pathogenic frameshift variant in exon 25 of *TSC2* provided additional molecular confirmation. Abdominal MRI also revealed a left renal hamartomatous lesion; the available imaging was insufficient to classify it definitively as an angiomyolipoma, and it was therefore not counted as a major diagnostic feature. Consequently, the patient was definitively diagnosed with coexisting WD and TSC. This dual diagnosis provides a more coherent explanation for the clinical course, particularly the recurrence of focal seizures during attempts to withdraw carbamazepine.

The management strategy for this patient required an individualized approach to each condition. Copper chelation therapy with penicillamine (750 mg/day) was continued for WD and was well tolerated. For TSC, because the seizures were medication-controlled and no other complication required immediate intervention, a surveillance plan was established according to TSC management guidelines (Northrup et al., 2021), including periodic neuroimaging and renal ultrasonography. At the 3-month follow-up, seizure control remained stable with no breakthrough events, and liver function tests were within normal limits. At 6 months, abdominal ultrasonography showed no increase in the size of the left renal hamartomatous lesion (maximum diameter 2.1 cm, unchanged from baseline). The patient and family received genetic counseling regarding the apparently *de novo TSC2* variant and the autosomal recessive inheritance of WD.

## 3 Discussion

WD and TSC are distinct disorders with limited overlap in their clinical features. Epilepsy affects approximately 80%–90% of individuals with TSC but occurs in only a minority (6.2%–8.3%) of neurologically affected patients with WD, in whom generalized tonic-clonic seizures predominate and seizure control is often achievable with conventional AEDs (Prashanth et al., 2010; Dening et al., 1988). In this patient, the focal seizures were controlled with carbamazepine 600 mg/day and were therefore not drug-resistant. The atypical clue was repeated seizure recurrence whenever carbamazepine was withdrawn despite effective copper management. WD primarily affects the basal ganglia and liver, whereas TSC is characterized by cortical and subependymal lesions and multisystem hamartomatous manifestations that can underlie focal epilepsy (Nabavi et al., 2022). The patient’s initial tremor, dysarthria, low ceruloplasmin, and response to chelation were consistent with WD; the later combination of recurrent focal seizures during carbamazepine withdrawal, characteristic cutaneous stigmata, subependymal nodules, and a probable cortical tuber supported an additional diagnosis of TSC. Although cutaneous changes may occur in WD(3), they are less specific than the TSC-associated skin findings observed in this patient.

The diagnostic process was complicated by the absence of genetic confirmation in 2009, when the WD diagnosis relied on clinical, biochemical, and reported imaging findings. Whole-exome sequencing later established the dual molecular diagnoses: the homozygous *ATP7B* variant explained WD, whereas the heterozygous *TSC2* variant confirmed TSC. This case illustrates diagnostic overshadowing, in which new manifestations are attributed to an established diagnosis (Jones et al., 2008). Here, the focal seizures were initially attributed to WD-related brain injury, delaying recognition of TSC. The delay likely reflected both diagnostic anchoring on the established WD diagnosis and the subtlety of the characteristic cutaneous findings, which were not recognized during earlier examinations.

Importantly, the diagnostic turning point preceded genetic confirmation and arose from a return to clinical fundamentals. New focal seizures after substantial improvement of WD, together with seizure recurrence whenever carbamazepine was tapered, prompted broader reassessment. Careful neurological and dermatological examination then identified facial angiofibromas, periungual fibromas, a shagreen patch, and hypomelanotic macules, reshaping the diagnostic trajectory. This case emphasizes that clinical incongruence should trigger renewed history-taking and physical examination even when an existing diagnosis is well established.

Notably, despite the presence of a predicted severe truncating variant in the *TSC2* gene (Eser et al., 2024), the patient presented with relatively mild multisystemic manifestations. The most plausible explanation is the well-recognized phenotypic variability of TSC, which can occur even among individuals with identical *TSC2* mutations. Experimental studies in unrelated systems link copper homeostasis with AMPK-mTOR and mitochondrial pathways (Guo et al., 2022; Li et al., 2023; Ramchandani et al., 2021), but these data do not establish a WD-TSC interaction, particularly after years of effective copper chelation. Any copper-related modification of the TSC phenotype therefore remains speculative and cannot be inferred from this single case.

Management requires parallel disease-specific care. For WD, lifelong therapy with chelating agents such as penicillamine or trientine, or zinc-based maintenance when appropriate, remains the cornerstone of treatment (European Association for the Study of the Liver, 2025). TSC requires multidisciplinary surveillance and treatment tailored to specific manifestations (Northrup et al., 2021). In this patient, seizures remained controlled with carbamazepine, and no TSC complication required immediate mTOR-directed therapy. Ongoing neurological, renal, pulmonary, dermatological, and other organ-specific surveillance remains important.

This report has several limitations. First, it is based on a single patient, limiting generalizability. Second, the original 2009 and 2012 neuroimaging and EEG materials were unavailable, limiting retrospective verification of the early course. Third, biological parentage was not independently confirmed by STR or equivalent testing; therefore, the TSC2 *de novo* evidence was conservatively down-weighted to PS2_Moderate. Finally, this case cannot establish a mechanistic relationship between WD and TSC, and natural phenotypic variability remains a more plausible explanation than a copper-mTOR interaction.

In conclusion, WD and TSC are genetically distinct disorders whose independent co-occurrence is expected to be extremely rare given their individual prevalences. This case does not establish a biological link between the two diseases. Its principal significance is clinical: an established diagnosis can create diagnostic overshadowing when subsequent findings are automatically attributed to the original disorder. When the disease course becomes incongruent—here, focal seizures recurring during attempts to withdraw AEDs despite effective copper treatment—careful re-examination, attention to multisystem signs, and appropriately expanded genetic testing can reveal a second diagnosis. The case therefore supports diagnostic reassessment rather than mechanistic speculation as the central teaching point.

## 4 Patient perspective

For many years, I struggled with hand tremors and difficulty speaking. After being diagnosed with Wilson disease, chelation therapy helped me a lot. Later I developed seizures. The medication controlled them, but the seizures returned when doctors tried to stop the medicine. During a new evaluation, the doctors found that I also had tuberous sclerosis complex. Knowing the full picture has helped me understand why continued follow-up is important. I am grateful that my case can help other patients with similar diagnostic problems. I understand the importance of sharing my story and give my consent for this publication.

## Data Availability

The datasets presented in this article are not readily available because of ethical and privacy restrictions. Requests to access the datasets should be directed to the corresponding author.
